# Poly(vinyl alcohol)/ZSM-5 zeolite mixed matrix membranes for pervaporation dehydration of ethanol and n-propanol

**DOI:** 10.55730/1300-0527.3622

**Published:** 2023-08-23

**Authors:** Ji-Ting WANG, Zhen HUANG, Ya-Tong ZHU, Si Ning WANG

**Affiliations:** Department of Packaging Engineering, Tianjin University of Commerce, Tianjin 300134, P.R. China

**Keywords:** Poly(vinyl alcohol), zeolite, mixed matrix membrane, alcohol dehydration, pervaporation

## Abstract

In this study, mixed matrix membranes (MMMs) composed of poly(vinyl alcohol) (PVA) and porous ZSM-5 zeolite are thoroughly investigated for concentrating alcohols of ethanol and n-propanol via dewatering pervaporation. The effects of the zeolite content (10–30 wt.%), feed composition (5–30 wt.% water), and feed temperature (50–90 °C) on the pervaporation flux/separation factor and component permeance/selectivity of these MMMs are examined in detail. These MMMs achieve higher separation efficiency and pervaporation flux than their pure PVA counterparts as expected, even if the dehydration results strongly depend on the pervaporation conditions. The disparity in pervaporation performances acquired for different alcohol solutions may be understood in terms of polarity and molecular size, which differ among these alcohol molecules. The PVA/zeolite MMM of 20 wt.% ZSM-5 zeolite content performs substantially stably at 60 °C for the feed with 80 wt.% alcohol while maintaining separation factors of 660 or 820 and total fluxes of 970 or 825 g/m^2^h for dewatering water/ethanol and water/n-propanol, respectively. Thus, our membranes appear to be technically feasible for practical alcohol dehydration uses.

## 1. Introduction

Alcohols are known to be very vital solvents and are thus extensively employed in laboratories and industries. However, alcohols can readily form azeotropic solutions with water moisture under atmospheric pressure, and subsequently, obtaining absolute alcohols from various aqueous solutions through traditional distillation processes may not be feasible [1]. Ethanol (ETA) [2] and n-propanol (NPA) [3] are reported to have respective compositions of 95.6 and 72.1 wt.% at 78.1 and 87.7 °C when forming an azeotrope with water under atmospheric pressure. Currently, the separation of azeotropic mixtures may be achieved by optionally using extractive distillation, homogeneous/heterogeneous azeotropic distillation, variable pressure distillation, and clean pervaporation technology [4,5].

Among the above-mentioned separations, pervaporation is gaining ever-increasing attention for separating solutions with close boiling points, azeotropic mixtures, and liquid systems with similar molecular sizes. Using this methodology, a selective permeable membrane is employed as a separating barrier through which some species can more selectively transport than others when subjected to a liquid mixture as the feed, leading to preferred pervaporation separation efficiency [6]. Among the various membrane materials, polymeric membranes have promising pervaporation performance in addition to excellent processability and satisfactory mechanical flexibility. Their pervaporation separation efficiencies may be further improved after dispersing inorganic porous particles into continuous polymer matrixes to form mixed matrix membranes (MMMs) [7,8].

Poly(vinyl alcohol) (PVA) is very popular polymer material for pervaporation dehydration because of its excellent hydrophilicity [9]. Thus, it is used here for making PVA-based MMMs. To date, many studies have been conducted on the improvement of PVA dehydration performance by incorporating different porous entities such as zeolite [10–13], metal organic frameworks [14–16], silica [17], sodium montmorillonite (Na^+^MMT) [18], carbon nanotubes [19,20], graphene [21,22], and other inorganic particles [23,24].

In our earlier studies [12,13], efforts were made to embed ZSM-5 and H-β zeolites with different contents into PVA membranes for isopropanol (IPA) dewatering. Our pervaporation results revealed that zeolite incorporation can effectively boost the separation performance of PVA membranes with great potential for performing IPA pervaporation dehydration. Thus, it will be very interesting to extend these membranes for dewatering ETA and NPA and then make a comparison to study how different alcohols affect membrane dewatering performances. Similar extension studies are rather limited but very helpful to consolidate and expand these MMMs for the dehydration of more alcohols. Therefore, two alcohols, namely ETA and NPA, are studied to comparatively evaluate the dehydration performances of pervaporative PVA/ZSM-5 zeolite MMMs of different zeolite loadings. A dope casting solution protocol is employed to prepare these MMMs and pervaporation dehydration tests are then carried out to examine the influences of zeolite content, feed concentration, and feed temperature on the dehydration separation of ETA and NPA aqueous solutions and compare the effects of alcohol type on membrane dehydration. In this process, our previous results for dewatering highly concentrated IPA aqueous solutions [12] are cited for comparison purposes. Besides studying pervaporation dehydrations, these MMMs are also investigated via characterizations such as X-ray diffractometry (XRD), Fourier transform infrared spectroscopy (FTIR), and swelling measurements.

## 2. Experimental

### 2.1. Materials

PVA with a degree of polymerization of 1750 and a degree of hydrolysis of 99% was purchased from Tianjin Guangfu Fine Chemical Co., Ltd. (Tianjin, China) along with fumaric acid (FA, 99.0%). ZSM-5 zeolite with a SiO_2_/Al_2_O_3_ molar ratio of 50 was obtained from Tianjin Nankai Catalyst Co., Ltd. (Tianjin, China). ETA (analytical grade, ≥99.7%) and NPA (analytical grade, 99.8%) were provided by Tianjin Kaitong Chemical Reagent Co., Ltd. (Tianjin, China). Zeolite was activated at 200 °C for 6 h before any use and the other materials were used directly without any treatment.

### 2.2. Membrane preparation

Pure PVA membrane and its zeolite-embedded MMM counterparts were fabricated by following a dope casting protocol [12,13] and the entire procedure can be briefly described as follows. Initially, 12 g of PVA and 1 g of FA were weighed using a balance and then added to a round-bottom flask fed with 88 g of distilled water, which was then heated via a water bath at 95 °C under agitation for 4 h, resulting in a PVA aqueous solution with a concentration of 12 wt.%. The solution was cooled to room temperature and kept statically for 12 h to completely remove any bubbles inside, followed by pouring the solution onto a clean glass plate to form a nascent membrane flattened with a casting blade. After drying at ambient conditions for 24 h, the membrane was placed in a muffle furnace and heated for 90 min at 160 °C for the crosslinking of PVA with FA, finally leading to a pure PVA membrane. For preparing the MMMs, the mass ratio of PVA to water was kept constant at 12:88. After the ZSM-5 zeolite was added to distilled water, the flasks were heated at 95 °C under stirring for 1 h, followed by the addition of 12 g of PVA and 1 g of FA under continuous stirring for 16 h. The PVA was fed stepwise into the doping solution to better disperse the zeolite particles and yield an apparently homogeneous doping solution. The MMMs were then obtained as described above. The content of ZSM-5 zeolite in the final MMMs was 10, 15, 20, 25, or 30 wt.%, and for convenience these are referred to as Z-10, Z-15, Z-20, Z-25, and Z-30, respectively. The PVA crosslinked with FA was called C-PVA while pristine PVA was referred to as P-PVA. The resultant PVA MMMs and their pure counterpart were found to have membrane thicknesses of 30–50 μm.

### 2.3. Characterizations of zeolites and membranes

For acquiring the porous features of ZSM-5 zeolite, a BSD-PM2 analyzer (Beijing Beishide Instrument Technology Co., Ltd., Beijing, China) was used to measure the N_2_ adsorption and desorption isotherms at –196 °C and the zeolite sample was degassed at 300 °C for 6 h before performing N_2_ adsorption/desorption analysis. FTIR analysis via a Brooke Alpha-H FTIR analyzer (Germany) was conducted on pure PVA and MMM samples in the wavelength range of 400–4000 cm^−1^ and 32 scans were taken for each sample with resolution of 1 cm^−1^. Analytical measurements of the crystalline structures were performed by XRD for ZSM-5 zeolite and all PVA-based samples using a Rigaku D/Max 2500V/PC X-ray diffractometer (Rigaku, Tokyo, Japan) and samples were examined in the 2θ range of 3° to 50° at a scanning rate of 4°/min.

Swelling tests were conducted on C-PVA, Z-10, Z-20, and Z-30 samples using a gravimetric method. Cut samples were weighed and placed in airtight bottles, which were then filled with a test liquid. After being kept at ambient conditions at 28 °C for over 24 h, the swollen samples were removed and reweighed after brushing off the residual liquid on the sample surface with a filter paper. The degree of swelling (*DS*) was calculated for each sample using the following equation [12,13]:


(1) $$DS(\%)=\frac{W_{2}-W_{1}}{W_{1}}\times 100\%$$

Here, *W*_2_ and *W*_1_ are the mass (g) of the swollen and dry membrane, respectively. The final *DS* value was acquired as the average of three independent determinations.

### 2.4. Pervaporation experiments

**Figure 1:**
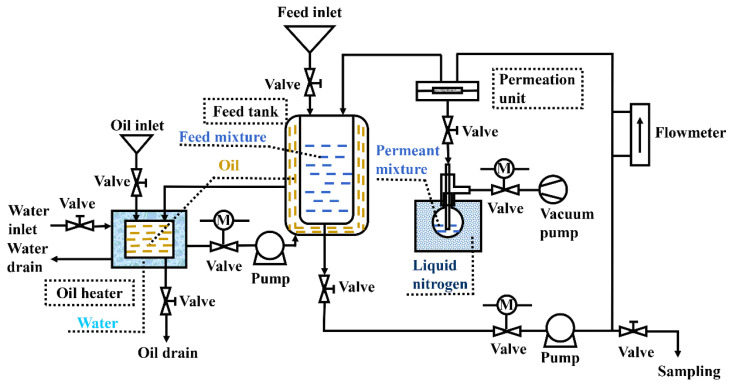
Schematic of experimental set up used in the present work for pervaporation.

The experimental setup for alcohol dehydration via pervaporation was similar to that used earlier [12,13] and its schematic is provided in Figure 1. The MMMs and C-PVA counterpart were examined in highly concentrated ETA and NPA aqueous solutions. For each test, over 4000 cm^3^ of aqueous solution was prepared with pure alcohol and distilled water to form an alcohol concentration of 75–95 wt.% and then fed into the feed tank, followed by heating the feed to a desired temperature of 50, 60, 70, 80, or 90 °C via the oil heating system. After properly mounting the membrane sample into the pervaporation unit cell, the feed was circulated at a flow rate of 4.0 × 10^4^ cm^3^/h. When the desired temperature was reached, the pervaporation test was started and the pressure downstream was maintained at about 100 Pa using a high-vacuum pump. The permeate vapor was collected for more than 30 min and condensed in a flask immersed in liquid nitrogen, and its composition was analyzed by a gas chromatography analyzer (GC 1100 Beijing Purkinje General Instrument Co. Ltd., Beijing, China). The GC analysis was conducted with an HP-5MS fused silica capillary column of 30 m × 0.25 mm with phenyl-methyl-siloxane as a stationary phase. While performing these tests, the column was kept in a GC oven heated from 180 to 280 °C at 10 °C/min and then held for 5 min. A thermal conductivity detector was employed at a helium flow rate of 2.0 mL/min. For each testing run, a volume of 1 μL was injected in a split mode with the injection and detector temperatures set at 210 and 250 °C, respectively. For each condition, triplicate measurements were done independently with acceptable errors of <5% for reproducing the flux and separation factor. In the meantime, sample mass was determined with an accuracy of ±0.1 mg using a digital balance.

The membrane pervaporation performances were evaluated using total permeation flux (*J*, g/m^2^h), separation factor (*α*), and pervaporation separation index (*PSI*), calculated as follows [12,13]:


(2) $$J=\frac{W}{S\cdot t}$$

(3) $$\alpha =\frac{Y_{w}/Y_{a}}{X_{w}/X_{a}}$$

(4) PSI=J·(α-1)

Here, *W*, *S*, and *t* are the permeate mass (g), the membrane working area of 13.85 cm^2^, and the pervaporation time (h), respectively. *X* stands for the component mass fraction in the feed and *Y* for the downstream permeate, and the subscripts *a* and *w* represent alcohol and water, respectively.

Apart from the flux and water/alcohol separation factor, data on water/alcohol permeances and selectivity are preferable as they can eliminate the effects of process conditions while reflecting intrinsic membrane properties. The permeance (*P*, g/m^2^h kPa) and selectivity (*β*) can be expressed as follows:


(5) $$P_{i}=\frac{J_{i}}{x_{i}\cdot \gamma_{i}\cdot p_{i}^{0}-y_{i}\cdot p_{pi}}\approx \frac{J_{i}}{x_{i}\cdot \gamma_{i}\cdot p_{i}^{0}}$$

(6) $$\beta =\frac{P_{w}}{P_{a}}$$

Here, *x*_i_ and *y*_i_ are the component *i* molar fractions in the feed and the permeate, respectively. *p*^0^ is saturated vapor pressure (kPa) at a specific temperature and *p*_p_ is the partial pressure (kPa) in the permeate, usually taken to be zero. Furthermore, *γ*_i_ is the component *i* activity coefficient in the feed, calculated using the Wilson equation [25,26].

## 3. 1. Results and discussion

### 3.1. Characterizations of zeolite and MMMs

#### 3.1.1. N_2_ adsorption/desorption

**Figure 2:**
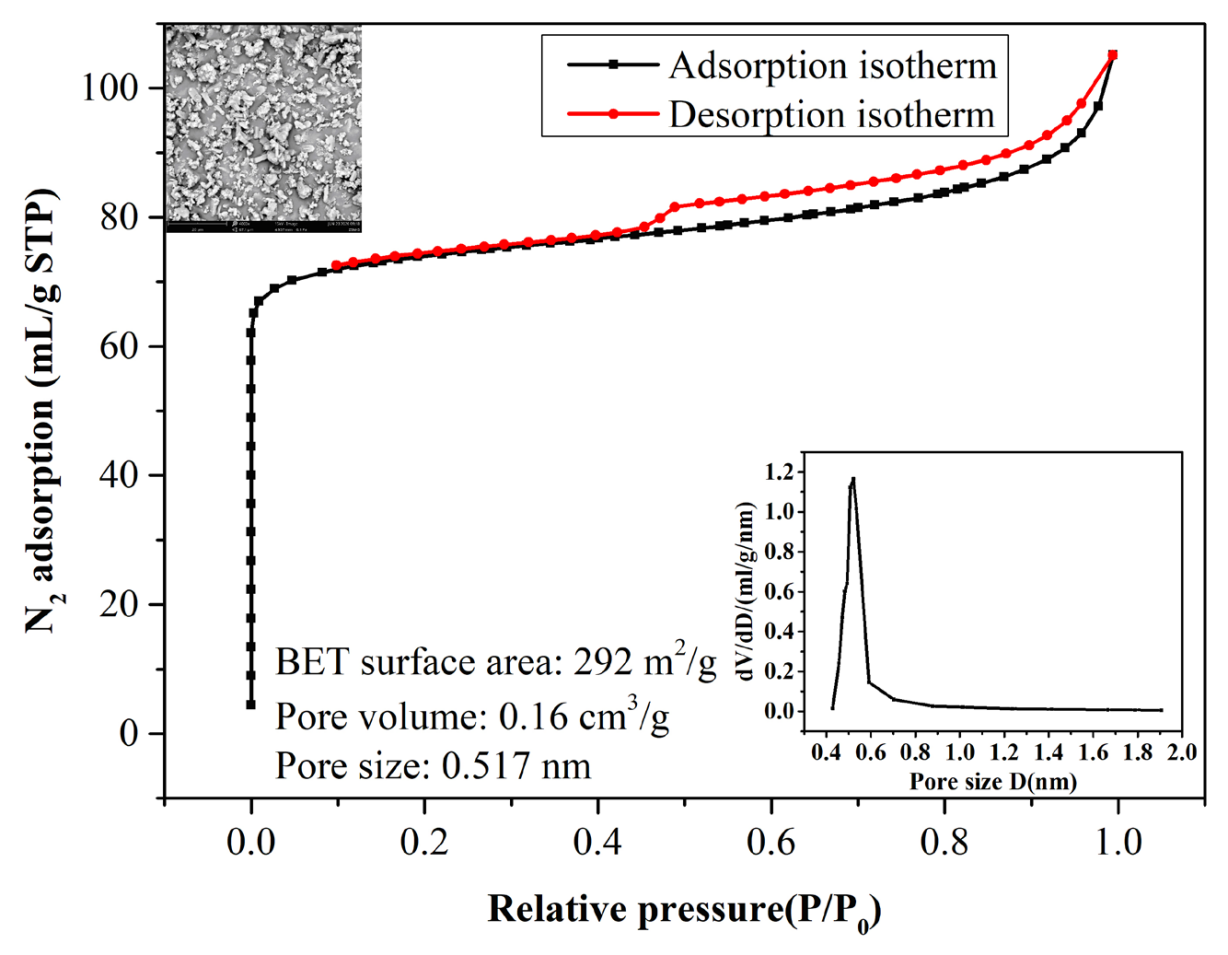
Nitrogen adsorption/desorption isotherms and SEM image of ZSM-5 zeolite.

Figure 2 shows the N_2_ adsorption/desorption isotherms measured for the ZSM-5 zeolite. As is clearly seen, it displays characteristic type I isotherms of microporous materials. That is to say, the adsorbed isotherm increases at a low relative pressure (P/P_0_ < 0.1) due to the quick filling of zeolitic micropores with N_2_ and afterwards the adsorption nitrogen amount increases gradually to the maximum as P/P_0_ approaches 1.0. Additionally, a hysteresis loop at high P/P_0_ is seen, likely due to the N_2_ filling of the interparticle mesopores formed in the zeolite. The specific surface area, calculated according to Brunauer–Emmett–Teller (BET) theory, and the total pore volume are 292 m^2^/g and 0.16 cm^3^/g, respectively. Following the Horvath–Kawazoe (H-K) method, the zeolitic pore size distribution curve was obtained and it was seen to peak at about 0.52 nm. These porosity results agreed very well with the porous features of ZSM-5 zeolite, which is an MFI-type zeolite with a pore structure intersected by a linear channel of 0.53 × 0.56 nm and zigzag channels of 0.51 × 0.55 nm [27]. The SEM image in Figure 2 reflects that the particle has a size of about 4 μm while somewhat serious particle agglomerations exist for the ZSM-5 zeolite.

#### 3.1.2. FTIR analysis

**Figure 3:**
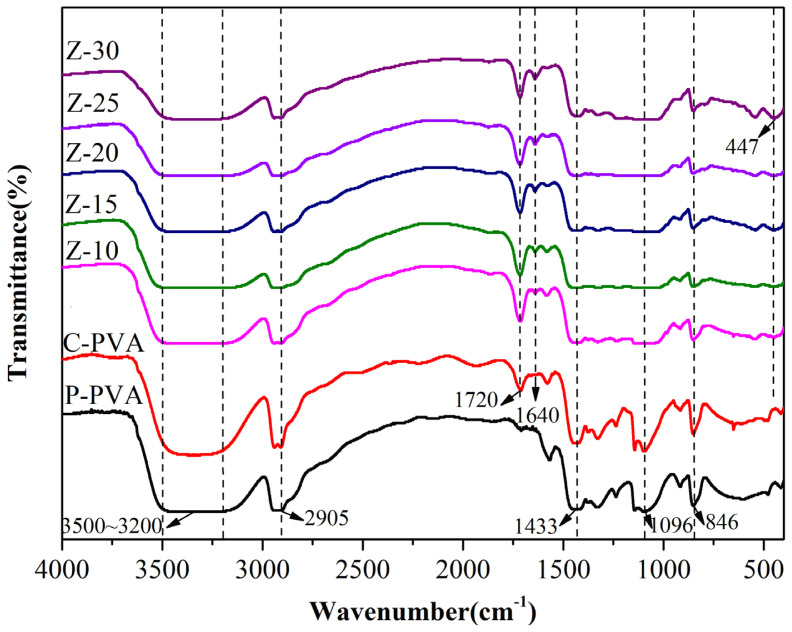
FTIR spectra of PVA/ZSM-5 zeolite MMMs and pure PVA membrane.

The FTIR analysis results acquired for the MMMs are presented in Figure 3. The FTIR patterns for these membranes are generally the same. The broad band around 3500–3200 cm^−1^ may be assigned to the presence of -OH groups in the MMMs and the pure PVA counterpart, since there are still a large number of -OH species in the PVA chains although the PVA might have reacted with the FA crosslinker. The bands at about 2905 cm^−1^ and 846 cm^−1^ may represent the respective symmetric vibration and deformation vibration of -CH groups while the band at 1433 cm^−1^ can be attributed to the bending vibration of -CH_2_. Furthermore, the band near 1096 cm^−1^ may be attributed to the stretching vibration of C-O [28], while the bands at 1720 cm^−1^ and 1640 cm^−1^ represent the stretching vibrations of C=O and C=C in the C-PVA sample, respectively [29]. Additionally, the multiple bands from 447 to 1100 cm^−1^ may be due to the stretching vibrations of Si-O-Si and Al-O in the zeolite [30]. In contrast, the bands at 1720 cm^−1^ and 1640 cm^−1^ are respectively not observed for P-PVA, but for C-PVA and its MMMs they are related to the stretching vibrations of the C=O and C=C groups due to the crosslinking reaction between PVA and FA [29].

#### 3.1.3. XRD analysis

**Figure 4:**
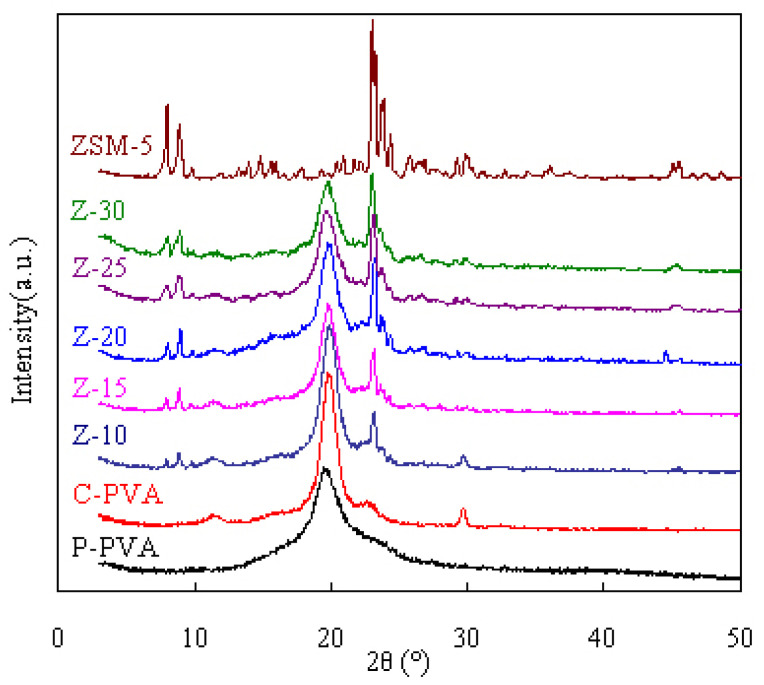
XRD spectra of PVA/ZSM-5 zeolite MMMs and pure PVA membrane.

Figure 4 presents the XRD analysis results obtained for ZSM-5 zeolite, pure PVA, and the MMM samples. As is clearly seen, the XRD spectra of the ZSM-5 zeolite demonstrated sharp peaks at 2θ = 7°–10° and 2θ = 22°, which are consistent with those reported in the literature [31]. As for PVA, a very broad peak appeared at about 2θ = 20° in its XRD patterns and that broad peak is still maintained after reaction with FA and filling with the ZSM-5 zeolite. However, the peak intensity is seen to decrease as the zeolite content increases in the MMMs. On the other hand, the characteristic peaks of the ZSM-5 zeolite are all observed in the MMMs while the peak intensities are found to increase with zeolite content.

#### 3.1.4. Swelling analysis

**Figure 5:**
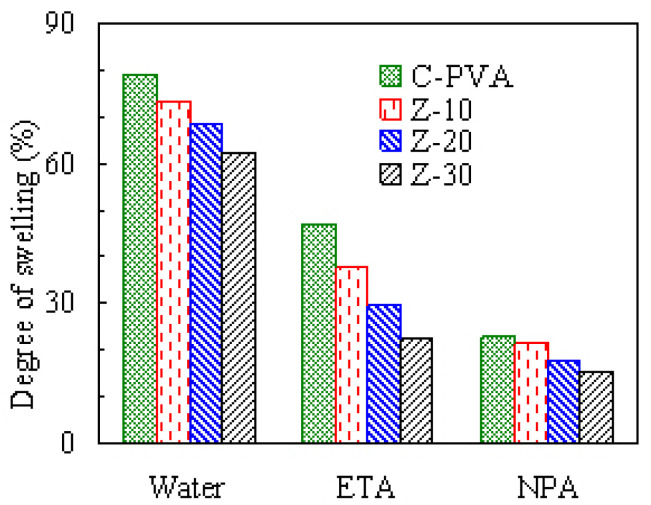
Swelling results (*DS*) of various PVA/zeolite MMMs.

Pristine PVA is known to be water-soluble and it can completely dissolve in hot water. Thus, it must be crosslinked to counteract water-soaking or else it may be unsuitable for dehydration pervaporation. The C-PVA and its MMMs were therefore studied here with swelling tests in water, ETA, and NPA, and the swelling results are shown in Figure 5. C-PVA is clearly seen to exhibit the highest *DS* value of 78.9% in water and this is probably due to the presence of many hydroxyl groups in the PVA chains, leading PVA to possess a strong affinity for water through hydrogen bonds. After embedding of the zeolite entities, the swelling of PVA was reduced substantially and the *DS* value declined by about 10% for the Z-10 MMM. When more zeolite was added, the membrane swelling was further compressed and the *DS* value decreased by 34% for the Z-30 MMM. The reason for this decreased swelling is probably related to the less hydrophilic and rigid features of the ZSM-5 zeolite as compared to the highly hydrophilic C-PVA, consequently leading to the MMMs withstanding water swelling and having lower *DS* values [32]. Similar swelling findings were observed for ETA and NPA, as well. However, the *DS* values in the three liquids for the same membrane were clearly different, decreasing in the order of water > ETA > NPA, which agrees very well with the polarity ranking in terms of dipole moment with dipole moment values of 1.82, 1.73, and 1.69 D for water, ETA, and NPA, respectively [33]. As might be expected, stronger polarity indicates the higher affinity between the polar liquid and the PVA membrane and then stronger swelling. As a consequence, the highest *DS* value is obtained for water, followed by ETA and finally NPA.

### 3.2. Pervaporation results of alcohol aqueous solutions

#### 3.2.1. Effect of zeolite contents

**Figure 6:**
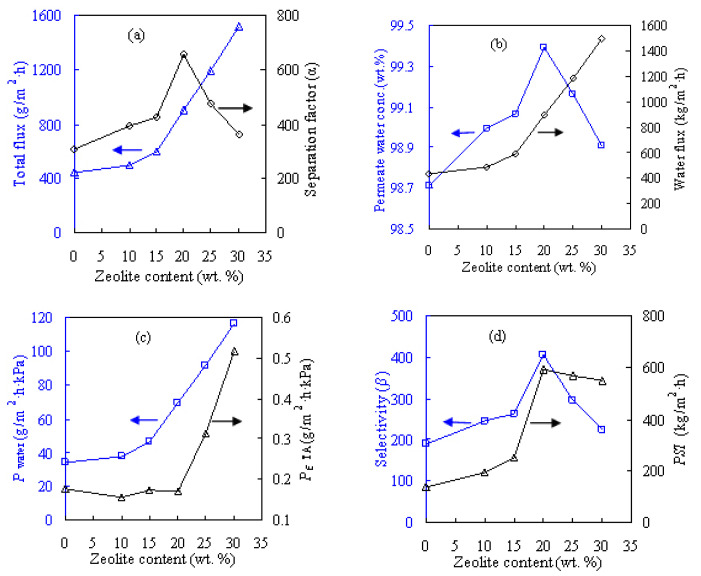
Effect of zeolite content on pervaporation performances of PVA/zeolite MMMs for feed with ETA content of 80 wt.% at 60 °C: a) total flux and separation factor, b) permeate water concentration and water flux, c) water and ETA permeances, and d) selectivity and *PSI*.

**Figure 7:**
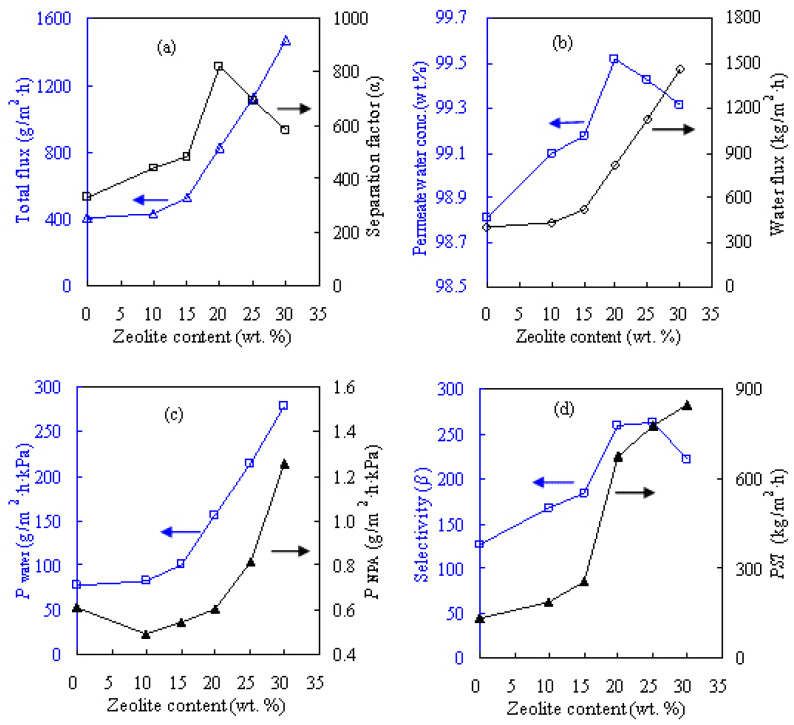
Effect of zeolite content on pervaporation performances of PVA/zeolite MMMs for feed with NPA content of 80 wt.% at 60 °C: a) total flux and separation factor, b) permeate water concentration and water flux, c) water and NPA permeances, and d) selectivity and *PSI*.

Figure 6 shows the influences of different zeolite contents on the pervaporation performances of the PVA-based membranes for the dewatering of the ETA/water solution and Figure 7 presents these results for the NPA/water solution. The zeolite content considered here varies from 10 to 30 wt.% while the pervaporation test is performed at 60 °C for the feed alcohol solution with 20 wt.% water. The zeolite addition altered the internal microstructure of the resultant membrane as well as the intrinsic membrane properties. As seen from Figures 6 and 7, these MMMs are water-selective and acquired appreciable separation efficiency, considerably outperforming the PVA counterpart in terms of both pervaporation flux and separation factor. At 20 wt.% zeolite loading, the water content in permeate side *Y*_w_ promisingly reaches more than 99.4 wt.% for dewatering the ETA and NPA solutions, as reflected in Figures 6b and 7b.

As shown in Figure 6 and 7, water permeance *P*_water_ and pervaporation flux *F* including the water and alcohol fluxes substantially increase as the zeolite content increases in the hydrophilic PVA membranes. These results can be explained by examining the zeolitic pore size and the molecule sizes of the liquids involved. It is known that ETA, NPA, and water molecules have kinetic diameters of 0.430, 0.469, and 0.296 nm, respectively. In contrast, the ZSM-5 zeolite has a relatively larger pore size of 0.517 nm, as shown in Figure 2. Thus, the addition of the ZSM-5 zeolite modifies the internal microstructures of the MMMs and then provides many porous passages for the penetrating molecules, subsequently favorable for both water and alcohol molecules to be readily transported through the MMMs. As such, more water/alcohol molecules can be transported across the membrane as the zeolite content increases, subsequently leading to the increase in pervaporation flux. For example, the unfilled PVA membrane for the ETA aqueous solution has flux of 442 g/m^2^h, and the flux is doubled to 901 g/m^2^h upon adding 20 wt.% ZSM-5. The maximum flux of 1516 g/m^2^h can be obtained when the content of ZSM-5 is 30 wt.%.

Looking at Figure 6, the alcohol dewatering separation factor *α*, selectivity *β*, and pervaporation separation index *PSI* are all found to increase with zeolite content, achieving peak values at zeolite loading of 20 wt.%. These values of *α*, *β*, and *PSI* tend to decline when the added zeolite content surpasses 20 wt.%, possibly due to the formation of nonselective defects around the zeolite particles in the parent PVA matrix at high zeolite loadings. Similar findings were also obtained for the NPA aqueous solution as shown in Figure 7 and the IPA aqueous solution reported previously [12]. Therefore, the PVA-based MMM with zeolite loading of 20 wt.%, referred to as Z-20, was considered for the subsequent dehydration investigations. However, the PVA-based membranes perform rather differently for the three alcohol solutions when examining the component permeance. As presented in Figures 6 and 7, the values of water permeance *P*_w_ obtained for the ETA/water and NPA/water mixtures respectively vary within ranges of 30–120 and 70–300 g/m^2^h kPa, while for IPA/water it varies in the range of 20–100 g/m^2^h kPa [12]. In the case of alcohol permeance *P*_a_, it is within the ranges of 0.1–0.6, 0.4–1.3, and 0.1–0.4 g/m^2^h kPa for ETA, NPA, and IPA, respectively.

Overall, for the same PVA/ZSM-5 MMM, total flux and penetrant fluxes appear to follow the sequence of ETA > NPA > IPA while dewatering separation factor *α* and selectivity *β* tend to follow the opposite sequence. These results may be attributed to the difference in molecular dimensions and polarity for the three alcohols. As addressed elsewhere [33], the kinetic diameter is reported to increase in the sequence of ETA < NPA < IPA, consequently contributing to the difference in pervaporation performances. In the meantime, the same polarity sequence was reported for the three alcohols [33], leading to different affinities within PVA-based membranes and different pervaporation results as discussed above. Unsurprisingly, similar dependency of dehydration performances on molecular size for some alcohol solutions have been reported recently [34–37]. For example, the dewatering separation factor of UiO-66-filled polyimide membranes was reported to decline in the sequence of ETA < IPA < n-butanol [34] or methanol < ETA < IPA [35], while the pervaporation flux followed the opposite sequence. Nevertheless, this dependency is not always strictly followed [38,39], as separation factors of IPA > n-butanol and flux of IPA < n-butanol may be observed.

#### 3.2.2. Effect of feed composition

**Figure 8:**
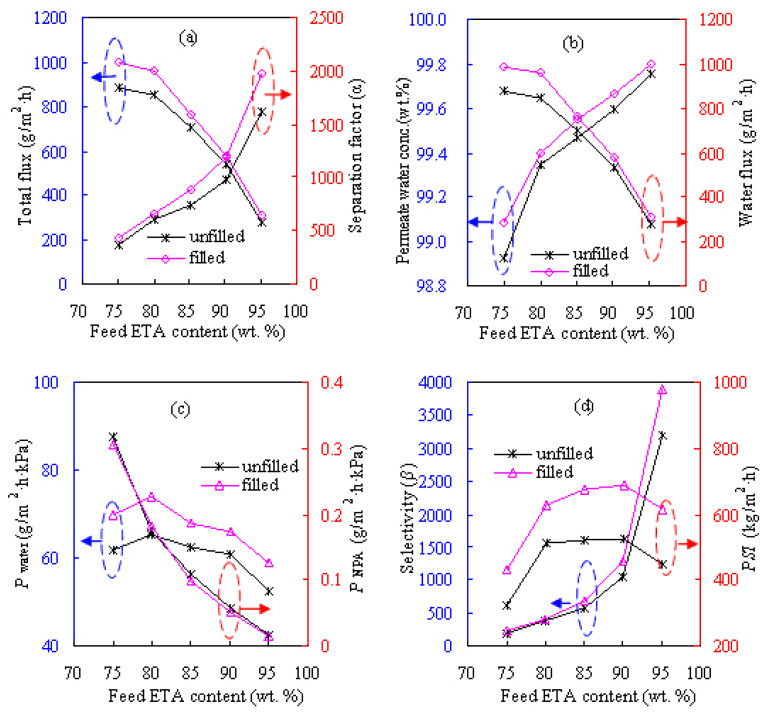
Effect of feed ETA composition on pervaporation performances of the Z-20 MMM at 60 °C: a) total flux and separation factor, b) permeate water concentration and water flux, c) water and NPA permeances, and d) selectivity and *PSI*.

**Figure 9:**
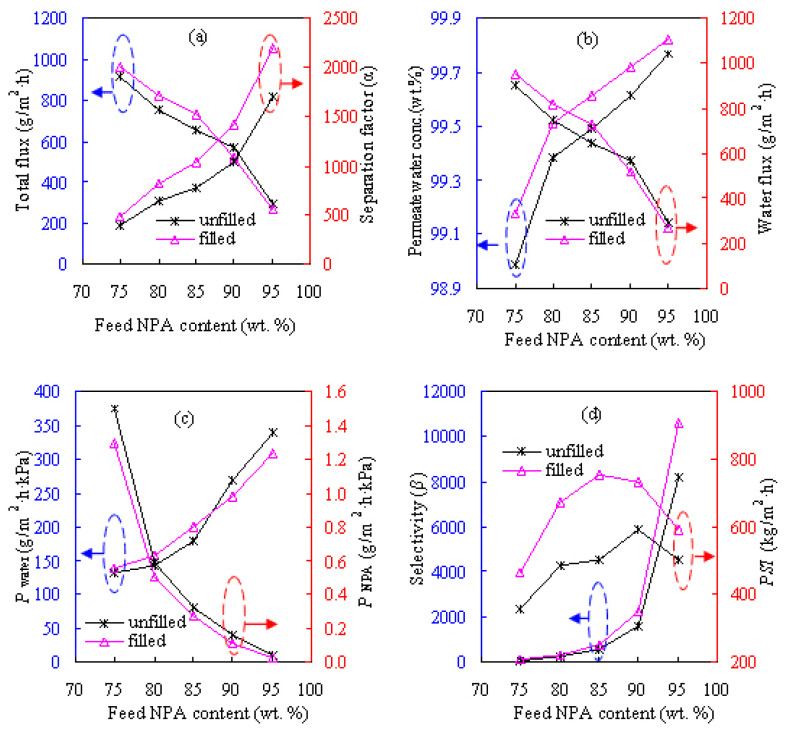
Effect of feed NPA composition on pervaporation performances of the Z-20 MMM at 60 °C: a) total flux and separation factor, b) permeate water concentration and water flux, c) water and NPA permeances, and d) selectivity and *PSI*.

Figure 8 shows the effects of feed alcohol composition of 75–95 wt.% on the pervaporation results of the Z-20 MMM for dewatering the ETA/water solution at 60 °C while Figure 9 provides the results for the NPA/water solution. As can be seen from Figures 8 and 9, *α*, *β*, and *Y*_w_ exhibit considerable increases as the feed alcohol content increases while *F* and *P*_a_ follow the opposite trend. These results can be attributed to the hydrophilic features of the water-selective PVA-based MMM. Such membranes might have stronger hydrogen-bonding capability with water than alcohol and may then form more water-swollen loose structures when contacting the water-richer feed. As a result, the components in the feed can be more easily and nonselectively transported through the membrane, bringing about enhancement in flux and decline in separation factor. When dewatering the NPA solution using the Z-20 MMM and its unfilled PVA counterpart, the total fluxes are found to decrease from 962 and 910 g/m^2^h for the 75 wt.% NPA feed to 269 and 295 g/m^2^h for the 95 wt.% NPA feed, respectively. Meanwhile, dewatering separation factor *α* is found to increase from 482 and 393 to 2197 and 1702, respectively. The same observations can also be made for dehydrating ETA as seen in Figure 8 and for IPA aqueous solutions [12].

In terms of the penetrant permeance over different feed compositions for the Z-20 MMM, as also seen from Figures 8 and 9, *P*_w_ varies in the ranges of 50–80 and 130–310 g/m^2^h kPa while *P*_a_ varies in the ranges of 0–0.4 and 0–1.3 g/m^2^h kPa for the ETA/water and NPA/water systems, respectively. As for IPA/water [12], *P*_w_ and *P*_a_ range within 50–65 and 0–0.3 g/m^2^h kPa, respectively. Promisingly, *Y*_w_ can be more than 99.5 wt.% for the three alcohol solutions, demonstrating outstanding alcohol dehydration efficiency.

For the three alcohols, the total pervaporation flux varies in the order of NPA < IPA < ETA for different compositions, whereas *α* and *β* vary in the order of NPA > IPA > ETA. The different results obtained for the three different feeds may be explained in terms of the coupling effect, i.e., the dragging force between water and alcohol, which is expectedly distinct for different alcohol–water pairs, as alcohol of higher polarity tends to exhibit a strong dragging force with water. Accordingly, the mutual dragging force may be better described as the pulling force of water molecules to alcohol and the backward traction force of alcohol molecules to water for hydrophilic dehydration of PVA membranes. In this way, the pulling force will render the transport of more alcohol molecules across the membrane, especially when the feed is richer in water. Thereafter, the membrane becomes less water-selective, consequently leading to a decline in dehydration efficiency. On the other hand, the backward traction force could retard the water permeation through the membrane and then reduce the water flux when alcohol is more plentiful in the feed. According to the trends of high polarity, it may be deduced that the coupling effect follows the sequence of ETA > NPA > IPA, therefore accounting for the different pervaporation results obtained for the three alcohol systems.

#### 3.2.3. Effect of feed temperature

**Figure 10:**
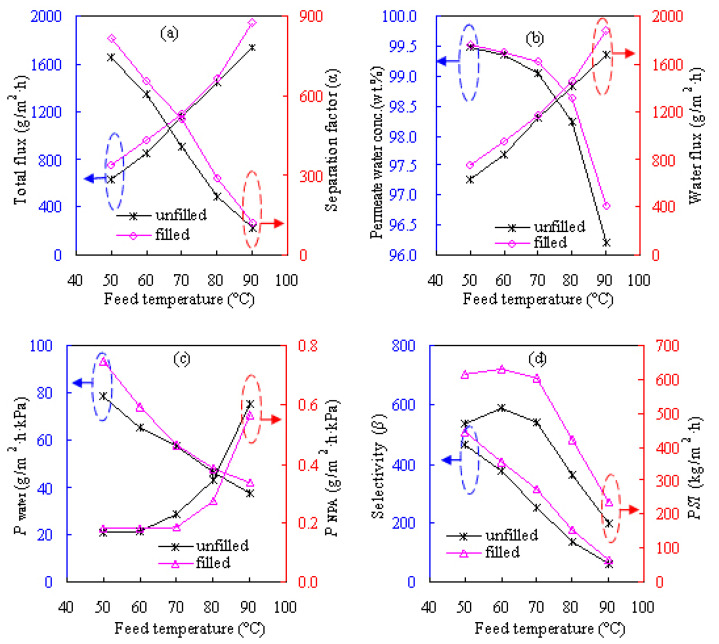
Effect of feed temperature on pervaporation performances of the Z-20 MMM for feed with ETA content of 80 wt.%: a) total flux and separation factor, b) permeate water concentration and water flux, c) water and ETA permeances, and d) selectivity and *PSI*.

**Figure 11:**
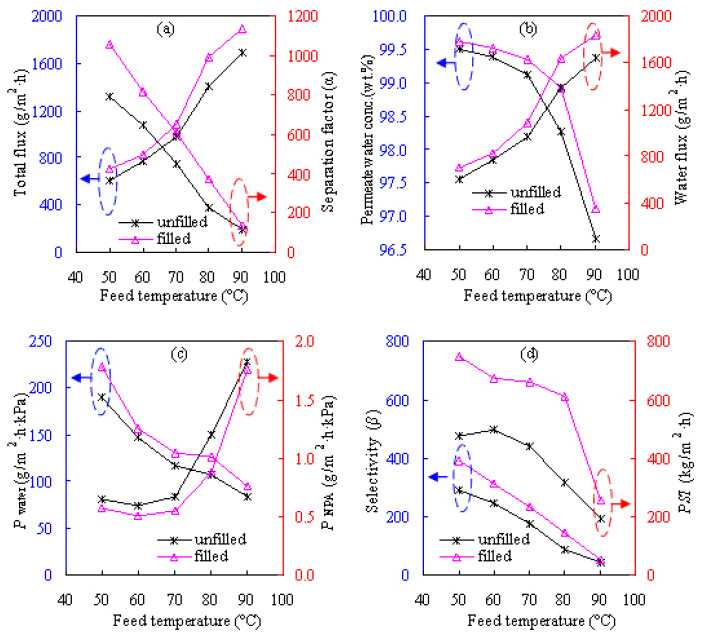
Effect of feed temperature on pervaporation performances of the Z-20 MMM for feed with NPA content of 80 wt.%: a) total flux and separation factor, b) permeate water concentration and water flux, c) water and NPA permeances, and d) selectivity and *PSI*.

Figure 10 shows the effects of feed temperatures of 50–90 °C on the dewatering performances of the Z-20 MMM for the ETA/water solution while Figure 11 provides these results for the NPA/water solution while an alcohol concentration of 80 wt.% is maintained in the feed. Clearly, *α* drops significantly, whereas *F* is boosted significantly as the feed temperature increases. For instance, at 50 °C, *F* and *α* are 754 and 710 g/m^2^h and 817 and 1055 for ETA/water and NPA/water, respectively. Meanwhile, at 90 °C, *F* and *α* become 1941 g/m^2^h and 122 for ETA/water and 1889 g/m^2^h and 135 for NPA/water, respectively.

The effect of feed temperature on pervaporation results may be understood from two perspectives. First, the PVA chains become more flexible and the membrane’s internal structure becomes microscopically looser as the feed temperature increases, and then the membrane’s free volume increases, leading to the transportation enhancement of the penetrating species. Second, water and alcohol can volatilize more easily and then they diffuse through the membrane more readily at higher temperatures. As a result, *F* and *P*_a_ increase significantly whereas α, *β*, *P*_w_, and *Y*_w_ all decline remarkably with an increase in feed temperature.

The effects of feed temperature on pervaporation fluxes and permeances can be approximated for the ETA/water and NPA/water systems based on the Arrhenius equation [40], which is mathematically expressed as follows:


(7) $$J=J_{0}\;\text{exp}(-\frac{E_{J}}{RT_{f}})$$

(8) $$P=P_{0}\;\text{exp}(-\frac{E_{P}}{RT_{f}})$$

Here, *J*_0_ and *P*_0_ are preexponential factors (g/m^2^h kPa), while *E*_J_ and *E*_P_ are the activation energy (J/mol) based on pervaporation flux and permeance, respectively.

Accordingly, the obtained activation energies are presented in Table 1 and the *E*_J_ values are all seen to be positive for water and alcohols. More precisely, the *E*_J_ value for ETA or NPA is much higher than that for water, suggesting that a higher energy barrier is demanded for ETA or NPA than water and therefore leading to satisfactory dehydration performances. On the other hand, much higher *E*_J_ values for the alcohols suggest that the alcohol partial flux is more dependent on temperature than the water partial flux, thereby causing the separation factor to decline with feed temperature. Similarly, *E*_p_ values were obtained for the ETA/water and NPA/water systems, and they were positive for alcohol permeance and negative for water permeance, as shown in Table 1. These results suggest that alcohol permeance will increase and the reverse will occur for water permeance, thus affecting the compression of dewatering selectivity with temperature.

### 3.3. Operation stability tests of alcohol dehydration pervaporation

**Figure 12:**
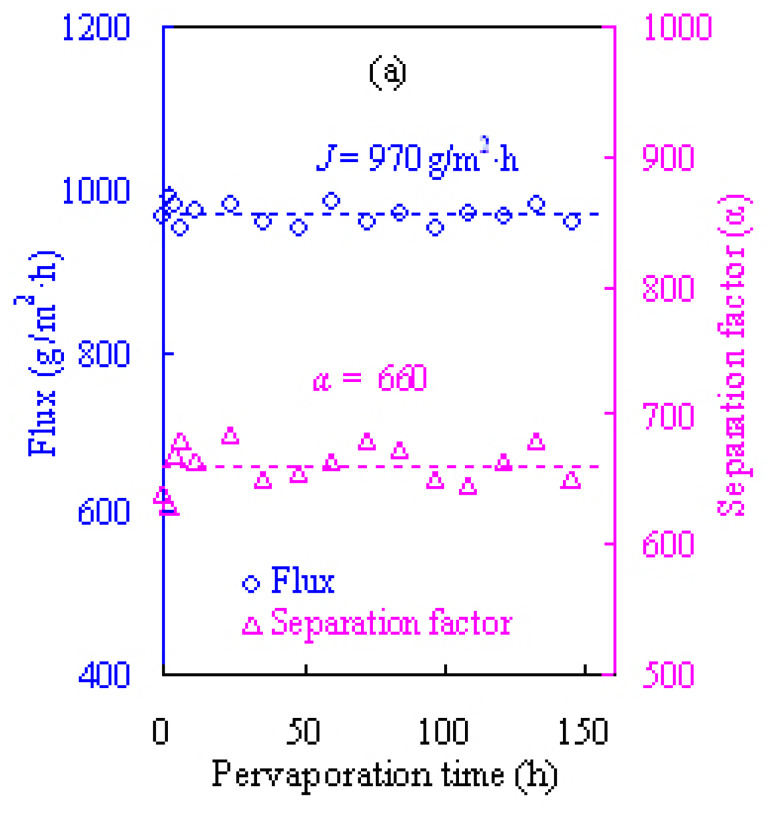
Operating stability of the Z-20 MMM for the dehydration of 80 wt.% ETA (a) and NPA (b) aqueous solutions at 60 °C.

For the purpose of practical dehydration applications, it was essential to examine the operation stability of the PVA/ZSM-5 zeolite MMMs developed in our work. The dewatering permeation evaluation was performed for 144 h on the Z-20 MMM at 60 °C with the 80 wt.% ETA and NPA aqueous solutions as the feed and the pervaporation results are shown in Figure 12. The total fluxes were observed to fluctuate initially and then hold constant at about 970 and 825 g/m^2^h with little variation for the ETA and NPA aqueous solutions, respectively. In the meantime, the dehydration separation factor was found to remain around 660 and 820 for the ETA and NPA aqueous solutions, respectively. The long-term results for flux and separation factor remained unchanged over 144 h for dehydrating ETA and NPA, indicating that the PVA/ZSM-5 zeolite MMMs have appreciable stability and significant separation potential to dewater various alcohols via pervaporation.

### 3.4. Comparisons with literature data

To further display their superior performances in dewatering alcohols, the pervaporation results of our PVA/zeolite MMMs were compared with literature data. Table 2 presents the ETA dehydration performances of recently developed pervaporative PVA-based membranes [15,16,18,19,21–24,41–56]. As seen in Table 2, the Z-20 MMM developed here has a water/NPA separation factor of 1188 and pervaporation flux of 581 g/m^2^h when dewatering the 90 wt.% ETA aqueous solution at 60 °C. These results confirm substantially higher performance compared to most PVA-based membranes under roughly similar pervaporation conditions. Clearly, the Z-20 membrane has outstanding dewatering performances as reflected by the *PSI* values. Therefore, it may be deduced from the results in Table 2 and Figure 12 that the MMM developed here has exhibited excellent pervaporation performances, and from a technical perspective, it can be practically used for the highly efficient dehydration of various alcohols.

## 4. Conclusion

In the present work, PVA MMMs filled with ZSM-5 zeolite of different contents were successfully prepared and characterized using a few different analysis methods. Pervaporation dehydration was performed for ETA and NPA aqueous solutions using these MMMs. Some conclusions may be drawn as follows:

1. The XRD results revealed that the continuous PVA matrix and dispersed zeolite particles still retained their respective crystalline features in the MMMs, while swelling tests showed that zeolite incorporation considerably compressed the membrane swelling and the swelling extent increased in the order of NPA < ETA < water according to polarity.
2. Zeolite embedding significantly altered the intrinsic pervaporation properties based on its porosity and hydrophilic features. The pervaporative PVA/ZSM-5 MMMs performed better than their parent PVA counterpart in terms of separation factor and total flux for dewatering the highly concentrated ETA and NPA aqueous solutions.
3. The MMMs displayed a continuous increase in total flux/water permeance for ETA and NPA aqueous solutions as the zeolite amount increased, while the dehydration separation factor/selectivity was found to increase until the zeolite amount reached 20 wt.%. In the meantime, the Z-20 MMM exhibited higher pervaporation flux/water permeance and lower separation factor/selectivity when dewatering at higher temperatures or higher feed water contents and vice versa.
4. The MMMs revealed distinct performances for dehydrating three different alcohol solutions, and the separation factor/selectivity and permeate water concentration values were found to increase in the order of ETA < NPA < IPA while the reverse was found for total flux and water permeances. This agreed very well with the differences in alcohol molecular diameter and polarity.
5. The PVA/ZSM-5 zeolite MMM of 20 wt.% zeolite loading developed here demonstrated outstanding pervaporation stability and superior separation performances for dehydrating ETA, NPA, and IPA compared to other studies. Thus, technically speaking, it can be applied for practical alcohol dehydration applications.

## Figures and Tables

**Table 1:** Calculated activation energies for C-PVA and Z-20 membranes.

Species	PVA		Z-20	

	*E*_P_ (kJ/mol)	*E*_J_ (kJ/mol)	*E*_P_ (kJ/mol)	*E*_J_ (kJ/mol)
Water	−17.70	21.98	−19.86	25.32
ETA	31.17	66.63	25.48	72.32
Water	−18.96	25.81	−18.81	25.30
NPA	26.53	72.37	26.83	72.66

**Table 2:** A comparison of ETA dehydration performances for pervaporative PVA-based membranes developed since 2015.

Membrane	Feed (wt.%)	T (°C)	Flux (g/m^2^h)	Separation factor	*PSI* (kg/m^2^h)	Ref.
PVA/CNTs	90	30	471	78	36	[41]
PVA/CNTs-COOH	90	30	395	662	261	
PVA/TiO_2_/CNTs	90	30	388	805	312	
PVA/Clay	90	50	380	459	174	[42]
PVA/ZIF-8	85	50	185	119	22	[43]
PVA/ZIF-8-NH_2_	85	50	158	148	23	
PVA/MIL-53-NH_2_	92.5	40	1600	4.5	6	[44]
PVA/MIL-53-NHCOH	92.5	40	965	13	12	
PVA/MIL-53-NHCOC_4_H_9_	92.5	40	1480	12	16	
PVA/ZIF-8	90	25	486	4725	2296	[45]
PVA/PETS	85	60	170	270	46	[46]
PVA/DEDPS	85	40	122	361	44	
PVA/APTES	85	40	102	247	25	
PVA/Silica	90	30	50	180	9	[47]
PVA/PAN	90	60	1736	9.7	15	[48]
PVA/C_3_N_4_/PAN	90	60	5915	19.9	112	
PVA/PES	90	60	908	9	7	
PVA/C_3_N_4_/PAN	90	60	4974	16.4	77	
PVA/ZIF-90	90	60	325	564	183	[49]
PVA/C_60_(C(COOH)_2_)_3_	70	50	127	775	98	[50]
PVA/PAN	90	75	2325	18.7	41	[24]
PVA/C_3_N_4_/PAN	90	75	4634	32.4	146	
PVA/C_3_N_4_-COOH/PAN	90	75	3598	40.7	143	
PVA/PDA-C_3_N_4_-COOH/PAN	90	75	2328	57.9	132	
PVA/UiO-66	90	30	48.2	41.6	2	[51]
PVA/UiO-66-OH	90	30	42.1	49.6	2	
PVA/UiO-66-(OH)_2_	90	30	46.3	46.3	2	
PVA/Ag	90	50	89	101.7	9	[52]
PVA/GO	90	40	137	263	36	[21]
PVA/GO	90	45	145	3095	449	[53]
PVA/Ti_3_C_2_T_x_	93	37	74	2585	191	[23]
PVA/CG	90	30	750	1425	1068	[22]
PVA/MA	96	50	112	202	23	[19]
PVA/CNT	96	50	271	111	30	
PVA/CNT-COOH	96	50	183	534	98	
PVA/MA/CNT-COOH	96	50	151	2158	326	
PVA/CS	96	25	1010	210	211	[54]
PVA/CS-P	96	25	1450	263	380	
PVA/CS-GA	96	25	1380	92	125	
PVA/CS-G	96	25	1500	92.2	137	
PVA/CS-SO_3_	96	25	1350	243.3	327	
PVA/Na^+^MMT	96	30	242.4	12.2	3	[18]
PVA/SNW-1	95	75	187	1501	281	[55]
PVA/UiO-66-(COOH)_2_	90	60	1242	1040	1290	[15]
PVA/UiO-66-(COOH)_4_	90	30	494	856	422	
PVA/UiO-66-(OH)_2_	90	30	485	699	339	
PVA/ZIF-8	90	25	685	4821	3302	[16]
PVA/APTES/ZIF‐90	85	40	85	552	47	[56]
PVA	90	60	537	980	526	This work
PVA/ZSM-5(Z-20)	90	60	581	1188	690	
